# Virus-Induced Gene Silencing Using Tobacco Rattle Virus as a Tool to Study the Interaction between *Nicotiana attenuata* and *Rhizophagus irregularis*

**DOI:** 10.1371/journal.pone.0136234

**Published:** 2015-08-20

**Authors:** Karin Groten, Nabin T. Pahari, Shuqing Xu, Maja Miloradovic van Doorn, Ian T. Baldwin

**Affiliations:** Department of Molecular Ecology, Max Planck Institute for Chemical Ecology, Hans-Knöll-Str. 8, 07745, Jena, Germany; Institute for Sustainable Plant Protection, C.N.R., ITALY

## Abstract

Most land plants live in a symbiotic association with arbuscular mycorrhizal fungi (AMF) that belong to the phylum Glomeromycota. Although a number of plant genes involved in the plant-AMF interactions have been identified by analyzing mutants, the ability to rapidly manipulate gene expression to study the potential functions of new candidate genes remains unrealized. We analyzed changes in gene expression of wild tobacco roots (*Nicotiana attenuata*) after infection with mycorrhizal fungi (*Rhizophagus irregularis*) by serial analysis of gene expression (SuperSAGE) combined with next generation sequencing, and established a virus-induced gene-silencing protocol to study the function of candidate genes in the interaction. From 92,434 SuperSAGE Tag sequences, 32,808 (35%) matched with our in-house *Nicotiana attenuata* transcriptome database and 3,698 (4%) matched to *Rhizophagus* genes. In total, 11,194 Tags showed a significant change in expression (p<0.05, >2-fold change) after infection. When comparing the functions of highly up-regulated annotated Tags in this study with those of two previous large-scale gene expression studies, 18 gene functions were found to be up-regulated in all three studies mainly playing roles related to phytohormone metabolism, catabolism and defense. To validate the function of identified candidate genes, we used the technique of virus-induced gene silencing (VIGS) to silence the expression of three putative *N*. *attenuata* genes: *germin-like protein*, *indole-3-acetic acid-amido synthetase GH3*.*9* and, as a proof-of-principle, *calcium and calmodulin-dependent protein kinase* (*CCaMK)*. The silencing of the three plant genes in roots was successful, but only *CCaMK* silencing had a significant effect on the interaction with *R*. *irregularis*. Interestingly, when a highly activated inoculum was used for plant inoculation, the effect of *CCaMK* silencing on fungal colonization was masked, probably due to trans-complementation. This study demonstrates that large-scale gene expression studies across different species induce of a core set of genes of similar functions. However, additional factors seem to influence the overall pattern of gene expression, resulting in high variability among independent studies with different hosts. We conclude that VIGS is a powerful tool with which to investigate the function of genes involved in plant-AMF interactions but that inoculum strength can strongly influence the outcome of the interaction.

## Introduction

Arbuscular mycorrhizal fungi (AMF) form symbiotic associations among most vascular land plant species and among fungi of the phylum Glomeromycota [1]. The fungi are obligate biotrophs that require photoassimilates from their host plants; these photoassimilates are received in exchange for mineral nutrients, particularly nitrogen, phosphorous, sulphur, zinc and water [2–4]. Additional benefits of AMF infection are enhanced defense against herbivores and pathogens [5,6]. Communication between the two partners starts before physical contact: the fungi recognize flavonoids and strigolactones, a novel class of phytohormones released by plant roots, which activate AMF branching [7–9], whereas fungal lipochitooligosaccharides (mycLCOs) and chitooligosaccharides stimulate calcium (Ca^2+^) spiking in root epidermal cells [10,11]. The Ca^2+^ oscillations, a central feature of the common symbiotic pathway, initiate the interaction of AMF and of nitrogen-fixing rhizobia with plants. The Ca^2+^ signal is assumed to be decoded by a Ca^2+^- and calmodulin-dependent protein kinase (CCaMK) whose expression is required for the development of a functional symbiosis [12]. In response to physical contact, the fungus develops an appressorium on the root surface; roots then develop the pre-penetration apparatus that enables the fungus to penetrate the root [13,14], which is followed by the intraradical colonization of the root by the fungus. Ultimately, arbuscules are formed within the root cortical cells and surrounded by a plant-derived periarbuscular membrane in which the main exchange of nutrients takes place (symbiotic phase, [15]).

The colonization of the root system by AMF is controlled by highly coordinated gene expression involving the host plant and the symbiont [16]. In the past, many high through-put transcriptomic studies using expressed sequenced Tags, suppression subtractive hybridization and microarrays for the transcriptome analysis identified plant genes that are involved in root colonization by different AMF species. Those studies were mainly performed with the whole root system of the legumes *Medicago truncatula* and *Lotus japonicus* [17–24], *Petunia* [25] and rice (*Oryza sativa*) [26]. More recent studies have used laser microdissection to identify genes expressed during different stages of the colonization process [27–30]. Arbuscule-containing cells strongly express—among many other genes—many genes encoding mycorrhiza-specific transporters; these transporters have been shown to be crucial for a functional symbiosis [31–33]. Little information, however, is available about fungal genes expressed in response to plant infection [34]. AMF-specific changes in gene expression can be a direct effect of mycorrhization or due to indirectly altered physiological conditions caused by AMF, such as improved phosphate nutrition or changes in phytohormone and metabolite levels [35,36].

Microarray analysis depends on the genes/cDNAs spotted on a chip and may miss genes expressed only under specific conditions, that is, conditions not considered for the chip. In contrast, the serial analysis of gene expression (SAGE) is a technique based on the finding that a short nucleotide sequence called Tag is specific for each gene. After massive parallel sequencing, the frequency of the Tags is proportional to the amount of the corresponding transcripts in the sample [37]. Using Blast searches, Tags can be specifically annotated to a gene. Long Tags, such as the development of 26 bp Tags for SuperSAGE, improve the specificity of the annotation process [38]. SuperSage has been used to elucidate the large-scale gene expression pattern of a non-sequenced organism after it was challenged with an effector [39,40].

However, it is difficult to draw solid conclusions about the importance of a gene for the interaction if only a change in expression is considered. A common approach for a functional gene analysis is the use of RNAi gene silencing [41]. The creation of stably transformed lines is highly time-consuming, whereas transient gene silencing using virus-induced gene silencing (VIGS) is a much faster approach [42,43]. The method takes advantage of a plant’s RNAi- mediated antiviral defense mechanism [44] and allows the silencing not only of plant genes but also of the genes of heterotrophs, e.g. of insect genes expressed in plants that are consumed by insects [45]. The plant-AMF interaction shares many similarities with a plant-heterotroph interaction, and host-mediated gene silencing in AMF has been demonstrated by using *A*. *rhizogenes* transformation [46].

*Nicotiana attenuata* Torr. ex Wats., a species native to the Great Basin Desert of southwestern USA (also known as coyote tobacco) is a member of the Solanaceae family. Its interaction with herbivores, floral visitors and root- and leaf associated bacteria has been extensively studied [47–49], but its interaction with AMF remains poorly understood [50,51]. Furthermore, only a few fungal genes essential for the interaction with plants are known [34,52–55].

Here we report the use of SuperSAGE libraries generated from RNA isolated from *N*. *attenuata* root samples, both those infected with *R*. *irregularis* (previously named *Glomus intraradices*, [56]) and those non-infected to study the gene expression changes involved in the plant-fungus interaction. An in-house trancriptome *N*. *attenuata* database [57] and the publicly available *Glomus intraradices* database (INRA Glomus database [34]) were used to obtain longer sequences corresponding to the 26 bp SuperSAGE Tags related to *N*. *attenuata* and *R*. *irregularis*. Based on the number of Tags that matched with one of the two databases, we conclude that about 10% of the sequenced Tags belonged to the fungal partner. Eleven up and downregulated *N*. *attenuata* and five *R*. *irregularis* SuperSAGE Tags were validated by qPCR. In order to study the function of selected genes and their effect on the symbiosis, we successfully silenced three *N*. *attenuata* genes by virus-induced gene silencing. We also attempted to use plant-mediated RNAi to silence fungal genes involved in this plant-heterotroph interaction, but the attempt did not succeed.

Taken together, our data show that a core set of plant genes is induced during plant-AMF interactions independently of the host species but that the overall global gene expression pattern varies among different studies. Although we found that tobacco-rattle virus mediated gene silencing is a useful tool for functional gene analysis in Solanaceaous species, host-induced gene silencing in the fungal partner was not successful.

## Material and Methods

### Plant material and inoculation with *Rhizophagus irregularis*

*Nicotiana attenuata* wild type plants (31^st^ inbred line of seeds originating from Utah, USA) were germinated on Gamborg B5 medium according to [58]. During germination, plants were maintained at 26°C in an incubator with an 11/13 h day/night cycle. For the SuperSAGE experiment, 10-day-old seedlings (incubated at 30°C) were transferred to 1-L pots containing 3-month-old leek plants (*Allium porrum Carentan 2*) as nurse plants. Pots contained either *Rhizophagus irregularis* inoculum (5% Amykor in expanded clay particles (2–4 mm), topped with sand (inoculated plants), or 5% autoclaved inoculum (twice at 121°C for 30 min) as control. For the validation of the SuperSAGE data, seedlings were transferred to 0.45-L pots containing *Rhizophagus irregularis* (10% Biomyc Vital, www.biomycvital.de, mixed with expanded clay particles, 2–4 mm, equivalent to Amykor) and 10% autoclaved inoculum for controls. For better drainage, the bottom of the pots was covered with large expanded clay particles (8–10 mm), and on top, the pots were covered with sand.

For both set-ups, seedlings were covered with transparent cups for the first week, and watered with distilled water as needed. Lights were switched on two days after transfer. After 7 days the cups were lifted and the seedlings were fertilized with 30 ml of 0.3 g Ferty B1 (Planta Düngemittel, Regenstauf, Germany, http://www.plantafert.de/), 0.6 g [Ca(NO_3_)_2_x4 H_2_O] per L every second day. Additional water was given as needed. All plants in the greenhouse were maintained at a 16/8 h day/night cycle with 26 to 31°C during day and about 20°C during night and 45 to 55% humidity, average temperature, light intensity, and humidity changed slightly over seasons.

For the virus-induced gene silencing (VIGS) experiment, 10- to 14-day-old seedlings were transferred to sand in Teku pots (propagation trays, 5.3 cm diameter, Pöppelmann, www.poeppelmann.com) with plastic nets, covered with transparent lids and fertilized with 0.21 g Ferty B1 (Planta Düngemittel, Regenstauf, Germany, http://www.plantafert.de/) and 0.47 g [Ca(NO_3_)_2_x4 H_2_O], 0.0536 MgSO_4_, 0.455 ml Fe-DTPA (diethylene triamine pentaacetic acid) per L. Agro-inoculation with VIGS vectors was done 20 days after transfer. The plants were covered with a black tray for 2 days to provide high humidity, allowing bacterial cells and their vectors to become established. As soon as the *PDS* (*phytoene desaturase*)-silenced plants showed bleaching of the leaves (after about 13 days), plants were carefully removed from the Teku pots with their plastic nets, and all roots outside the nets were excised. The nets were transferred to 1-L pots containing *Rhizophagus irregularis* inoculum (10% Biomyc Vital mixed with expanded clay particles, 2–4 mm), and large expanded clay particles at the bottom and sand on the top of the pots as described. The first week after transfer, plants were watered only as needed and then fertilized with 30 ml of 0.3 g Flory B1 and 0.6 g [Ca(NO_3_)_2_x4 H_2_O] per L every 3 days, and additionally watered as needed. In a second experiment, plants were transferred to 1-L pots containing freshly cut leek roots grown on *Rhizophagus irregularis* inoculum for 48 days before starting the experiment. Fertilization was carried out as described above.

### Harvesting of root samples

Root samples for the generation of SuperSAGE data were harvested when they had a similar rosette size, ranging from 4.3 to 5.8 cm (average 4.9 cm). The first plants were harvested 19 days after transfer (only non-infected plants) and the last plants (mainly infected plants) five days later. In total, 10 plants were harvested for each library.

For the validation of selected SuperSAGE Tags by qPCR, we conducted a kinetics analysis, harvesting roots weekly in order to find the time-point when the root colonization of plants was most similar to the root colonization in samples used for the SuperSAGE analysis. Harvest took place when both infected and non-infected plants had reached almost the same size. The first plants were all harvested after 14 days, and the longest leaf was on average 1.77 cm long; the next set was harvested between 20 and 24 days (the average length of the longest leaf 3.13 cm), then between 28 and 32 days (average stalk length 12.5 cm), followed by 35–38 days (average stalk length 26.11 cm) and the last ones between 49 and 51 days (average stalk length 42.5 cm). For the first two time-points, the roots of two plants were pooled to obtain sufficient material.

For the VIGS experiments, plants were harvested 28 and 35 days after transfer to inoculum, and the fresh mass of roots and shoots was determined. Only the roots growing outside the plastic net and not in the large expanded clay particles were used for analysis, and carefully cut. For all experiments, roots were washed to remove any attached expanded clay particles or sand, carefully dried and cut into pieces of about 1 cm and mixed. A part of the root sample was stored in root storage solution (99% ethanol and 60% acetic acid in the ratio of 3:1) for staining. The remaining root sample was immediately frozen in liquid nitrogen and kept at -80°C until further use.

### Root fungal colonization rate

To determine fungal colonization rates, we harvested root samples at the time-points indicated in the text. Before staining, the storage solution was removed, washed with distilled water twice, cleared with 2% KOH for 5 min at 95°C, washed with distilled water, acidified with 5% HCl, washed with distilled water and stained with 0.05% Trypan blue in lactic acid:glycerol:water (1:1:1) [50]. The colonization rate was estimated with the gridline intersection method [59]. A total of 100–150 visual fields at 200x magnification were observed, and the number of hyphae, arbuscles and vesicles were recorded.

### RNA extraction, SuperSAGE analysis and qPCR

Approximately 100 mg of ground root sample was used for total RNA extraction according to [60]. To generate the SuperSAGE libraries, 10 RNA samples each of infected and non-infected roots were mixed and sent to GenXPRO GmbH (Frankfurt am Main, Germany), which isolated mRNA from total RNA by using the mRNA Purification Kit (Amersham Biosciences) and generated the libraries.

For the confirmation of the SuperSAGE data, we selected 16 differentially expressed Tags, 6 up-regulated *N*. *attenuata* Tags, 5 down-regulated Tags and 5 up-regulated *R*. *irregularis* genes. In order to obtain longer sequence information for primer design, we used the transcript sequence of the Tags’ BLAST hits. To avoid a nutrient drain due to the nurse plants, we used dry inoculum on expanded clay particles instead of nurse plants; nurse plants were used to generate the SuperSAGE libraries. Reverse transcription (RT) was done in 96-well microtiter plate using oligo (dT) and Superscript II reverse transcriptase according to the manufacturer’s instruction (www.invitrogen.cow). Real-time qPCR was performed on a Mx3005P qPCR system (Stratagene, Santa Clara, CA, USA, http://www.stratagene.com) with qPCR Core Kit for SYBR Green I (Eurogentec, Seraing, Belgium, http://www.eurogentec.com) following the manufacturer’s instructions. For all *N*. *attenuata* primers, a normal 2-step PCR (95°C for 10 min, 40 cycles of 95°C for 15 s and 60°C for 1 min) was carried out; the exception was *Nicotiana attenuata*-specific phosphate transporter-4 (*NaPT-4*, [51]) and *R*. *irregularis* specific primers: these primers were amplified with 3-step qPCR at 57°C and 60°C, respectively (95°C for 10 min, 40 cycles of 95°C for 15 s and 60 /57°C for 30 s, 72°C for 30 s), and putative *R*. *irregularis* ATPase 3 with normal 2 step qPCR at 57°C. A complete list of primers is available in Table A in S1 File. Melting curves were performed for all primers to rule out non-specific amplification. For the standard curve, a dilution series of cDNA of AMF-infected roots (six progressive 1:3 dilutions, including an undiluted sample of infected roots) and blank (MilliQ water) was established. For the normalization of cDNA concentrations, *actin* transcript levels [61] were initially determined, but due to variability between plants, *Nicotiana attenuata* specific *elongation factor alpha-1* was used as a reference gene [39,46,61].

### Sequence homology alignment of SuperSAGE data and Gene ontology (GO) annotation

SuperSAGE Tags provided by the GenXPRO GmbH (Frankfurt am Main,Germany) were cleaned by removing Tags with a high number adenosine bases (poly-A’s) and by removing singletons. The workflow is shown in S1 Fig. BLAST searches were carried out using the BLASTN algorithm with all Tag sequences against our in-house 454-*N*. *attenuata* transcriptome sequencing database [57] and the publicly available *Glomus intraradices* sequencing database (http://mycor.nancy.inra.fr/IMGC/GlomusGenome/blast3.html). Statistical analysis of differentially expressed tags was calculated according to [62]. For fold-change (FC) calculations, the libraries were normalized to 1,000,000 tags, and the FC for each tag was calculated by dividing the number of tags in the normalized non-infected library (-AMF) by the number of tags in the normalized infected library (-AMF vs +AMF). Tags absent in one of the libraries (Tag count = 0) were set to 1 for calculation. For protein and gene ontology (GO) annotations of differentially expressed Tags and for primer design, only Tags with a *P*-value <0.05 and a > I2I log2 change between infected and non-infected roots were used. The Blast2GO software (https://www.blast2go.com/) was used to predict gene functions based on gene ontology (GO) categories [63].

### Virus-induced gene silencing (VIGS)

**Fig 1:**
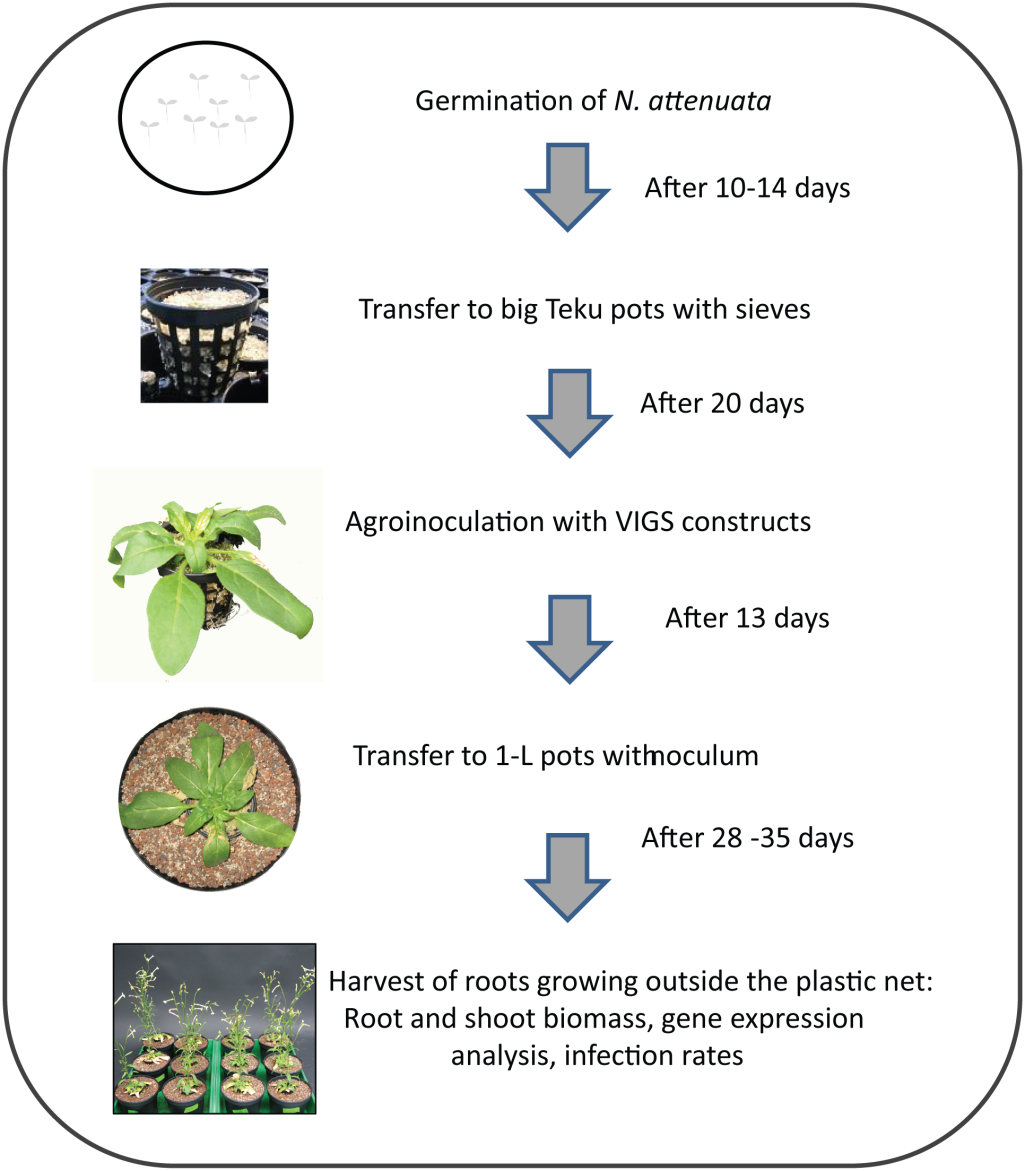
Experimental set-up for virus-induced gene silencing experiments to study the interaction between *Nicotiana attenuata* and arbuscular mycorrhizal fungi.

In order to analyze the role of selected putative genes involved in the interaction between *N*. *attenuata* and *R*. *irregularis*, we created vectors to silence two plant genes (*germin-like protein*, *putative indole-3-acetic acid-amido synthetase GH3*.*9*) and two fungal genes (*vesicle-associated membrane protein 7B*, *plasma membrane ATPase3*)–both of which were highly up-regulated in response to mycorrhizal infection—using tobacco rattle virus for gene silencing (VIGS) [42,43]. To test whether our approach was successful, we also created vectors for silencing a *calcium and calmodulin-dependent protein kinase*, a gene whose expression has been shown previously to be essential for the interaction between different plant species and mycorrhizal fungi [64,65], and a *Rhizophagus irregularis*-specific gene, *monosaccharide transporter 2* (*MST2*), which was successfully silenced in *Medicago truncatula* roots using *Agrobacterium rhizogenes* for transient gene silencing [46]. Table B in S1 File lists the genes and primers used for the construction of the VIGS vectors. Additionally, the pTVPD vector, harboring a part of the sequence of a phytoene desaturase (PDS), was used to monitor the progress gene silencing [43]. The construction of the VIGS-vectors and the inoculation of the plants were performed according to the published protocol [66], though the experimental set-up was adapted as shown in Fig 1. The main difference is that plants were grown on sand in larger Teku-pots and only transferred to 1L pots when pTVPD-treated plants showed bleaching as marker of successful gene silencing. At this stage, all roots growing outside the net that was used for planting were cut immediately before transfer to 1L pots to ensure that roots growing into the 1L pots were silenced in the gene of interest.

### Statistical Analysis

All statistical analyses were performed with the R statistical package (http://www.r-project.org/). Significance was assessed by one-way ANOVA followed by Tukey’s HSD if the differences were significant; values of *P* < 0.05 were considered statistically significant.

The SuperSAGE data were deposited in the Short Read Archive public domain under the accessions SRX1000071 for the AMF infected roots and SRX1000116 for the controls.

## Results

### Characterization of the SuperSAGE libraries from *Rhizophagus irregularis* infected and non-infected *Nicotiana attenuata* roots

In order to find out which genes are specifically regulated during the interaction of *N*. *attenuata* with *R*. *irregularis*, we generated SuperSAGE libraries from *R*. *irregularis*-infected and non-infected roots of size-matched plants. After eliminating incomplete reads, twin-ditags, and ditags without complete library-identification DNA linkers, sequencing resulted in 5,264,218 Tags, comprising 1,666,059 for the non-infected roots and 3,598,159 for the infected roots. The frequency distribution of these Tags showed that the number of copies in groups with low and average levels of Tags (≤ 1,000 copies million^-1^) represented 99.8% of the Tags, whereas groups with high and extremely high levels of Tags (>1,000 copies million^-1^) represented only 0.20% (Table C in S1 File).

After multiple adenosine bases were removed to rule out the possibility of sequencing errors, 92,434 Tags remained, and these were used for all down-stream analyses (S1 Table). For gene annotation, a basic local alignment (BLASTN) algorithm was performed with the 92,434 Tags against the in-house *N*. *attenuata* 454 transcriptome sequencing database [57]. Among these Tags, 32,808 (35%) showed a perfect match (26/26) with genes of the transcriptome database, whereas 3,698 (4%) of the Tags matched perfectly (26/26) with the publicly available *R*. *irregularis* genome sequencing database [34] (Table 1). Thus, about 10% (3,698/36,506) of the annotated Tags were from the fungal partner. When we used less stringent annotation parameters, such as 24/26 and 20/26 bp match, 44.5% (41,172) and 51.3% (47,480) of Tags could be annotated (Table D in S1 File).

**Table 1:** Annotation of SuperSAGE Tags using the in-house *N*. *attenuata* 454 transcriptome database [57] and the publicly available *Rhizophagus irregularis* sequencing database [34].

No. of Tags matching with *N*. *attenuata* 454 transcriptome sequencing database (26/26)	No. of Tags matching with *R*.*irregularis* sequencing database (26/26)	Unmapped
32,808 (35%)	3,698 (4%)	55,928 (61%)

Tags with a *P*-value of less than 0.05 and at least a 2-fold change were considered as significantly up- and down-regulated and were used to characterize SuperSAGE Tags.

From 11,194 Tags that significantly changed in response to infection, 4,729 (43%) Tags were up-regulated and 6,465 (57%) Tags were down-regulated. The vast majority of up- (4,275) and down-regulated (3,550) Tags were in the range of a two- to five-fold change; 89 Tags and 9 Tags were more than 100-fold up- and down-regulated in response to infection, respectively (S2 Fig).

**Fig 2:**
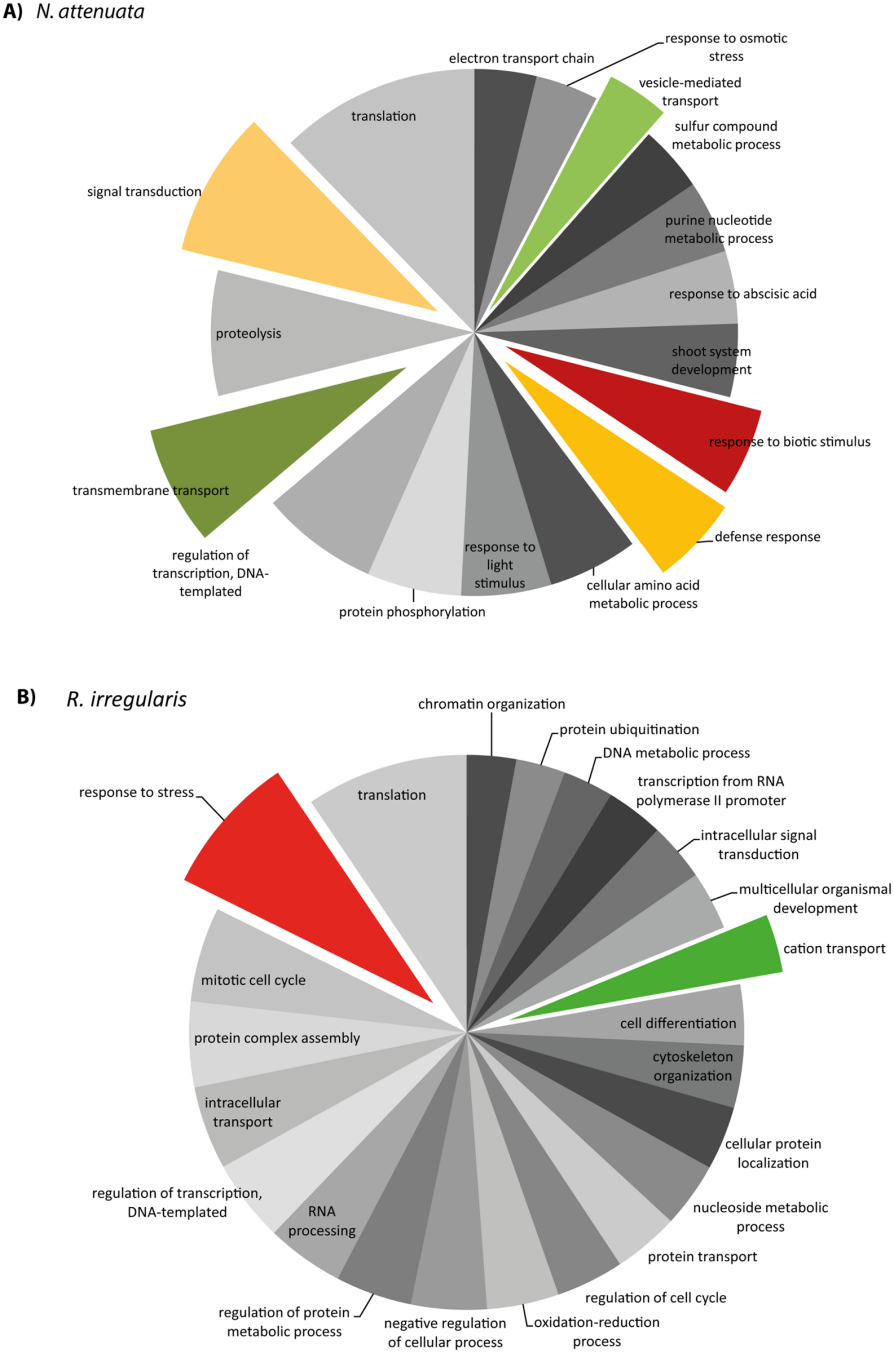
Gene ontology analysis showing node scores based on combined graphs of Tags matching with *N*. *attenuata* (A) and *R*. *irregularis* (B) genes. The classification of the genes was performed according to the gene ontology term "biological process". Only Tags with a significant (p≤0.05) log2 fold change >|2| were considered. The cut-off for node scores is 20.

Gene ontology analyses were performed to obtain further information about the gene function of differentially expressed Tags. Only Tags that mapped *Nicotiana attenuata* and *R*. *irregularis* genes and showed at least a log2 expression change >I2I were considered (*P* < 0.05). We used this strict cut-off to minimize the number of putative false-positives. Among the most prevalent GO biological processes (level 3) for *N*. *attenuata*, more than 50% of the sequences were classified as relating to cellular and metabolic processes. Interestingly, the overall pattern of gene functions regulated in response to infection was similar for both *R*. *irregularis* and *N*. *attenuata* (data not shown). However, when GO functions were compared based on node scores [67], functions related to signal transduction, transport and defense showed a high score for the plant partner (Fig 2), whereas for the fungal partner mainly basic cellular functions scored high, but also functions related to stress and cation transport (Fig 2B).

We compared our gene expression data with data from two other recent global gene expression analyses using AMF-infected tomato [68] and *Medicago* roots [29]. The comparison revealed 18 genes that were strongly up-regulated in all three studies (S2 Table). These genes cannot be related to a single function in the cell but are related to different functional groups. Remarkably, for all three studies, an *auxin-response protein*, *GA20ox* and three transcription factors as well as a *nitrate transporter* and *proteinases*, including a *subtilisin-like proteinase* were strongly regulated. In general, infected tomato roots showed more overlap with *N*. *attenuata* (additional 30 Tags with putative similar functions) than with *Medicago* (additional 15 Tags). It has to be noted that our comparison does not take into account if different subgroups of a gene family or subunits of a large protein were activated by AMF in the three studies. We also compared putative fungal Tags significantly expressed in *N*. *attenuata* and tomato, and the functional description of 51 annotated Tags overlapped (S3 Table). Many of these genes have rather general functions in cells, such as *actin*, *60* and *40S ribosomal proteins*, *histones* and *ubiquitin*. Others are related to N- and sugar metabolism (e.g. *glutamine synthase*, *fructose-bisphosphate aldolase*, *fructose-bisphosphate class II*, *triosephosphate isomerase*), indicating a high metabolic turnover within the fungal partner.

### Validation of the SuperSAGE data by qPCR

For the validation of the SuperSAGE data, we conducted a time-course analysis to determine the time-point when plants achieved infection rates similar to those in the roots used for the SuperSAGE analysis. The amount of infection structures counted based on microscopic observations and the expression of *N*. *attenuata phosphate transporter 4* gene (*NaPT4*) as a marker of arbuscule formation [31] increased with time (S3 Fig), and the values obtained in roots 35 days after inoculation matched best with the values in the roots colonized with fungus that were used for the SuperSAGE libraries (Table E in S1 File). We also tested gene expression of two other arbuscule-specific P-transporters from *N*. *tabacum NtPT3 and NtPT5* [69]. Their expression also corresponded with arbuscule formation, but correlation was less pronounced (data not shown).

**Fig 3:**
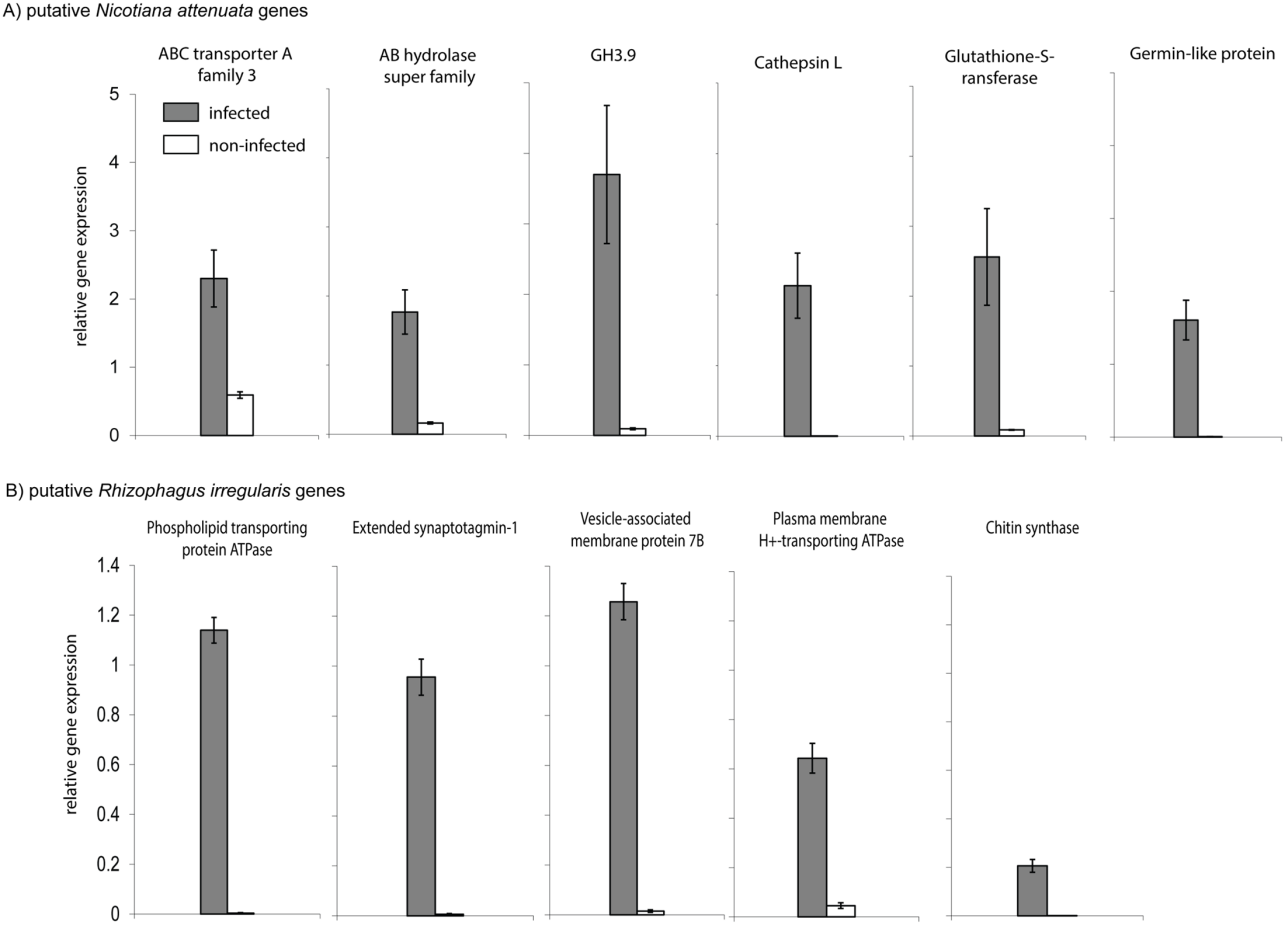
Validation of significantly up-regulated SuperSAGE Tags annotated to *N*. *attenuata* (A) and *R*. *irregularis* (B) by qPCR. Relative transcript levels of selected plant (A) and fungal (B) Tags significantly up-regulated (*P* < 0.05) after the infection of *Nicotiana attenuata* by *Rhizophagus irregularis*. qPCR analysis was carried out on root samples harvested 35 days after inoculation. *Elongation factor-1 alpha* was used as a reference gene for normalization. Transcript levels were analyzed in four biological replicates ((+ se, N = 4).

We used genes known from other plant-AMF interactions and genes that have not been described earlier for validation. The up-regulation of the probable *N*. *attenuata* genes *ABC transporter A*, *putative indole-3-acetic acid-amido synthetase GH3*.*9 (GH3*.*9)*, *capthepsin L*, *glutathione-S-transferase (GST)*, *germin-like-protein (GLP) and AB hydrolase* could be confirmed by qPCR (Fig 3; Table F in S1 File). Similarly, the putative *R*. *irregularis*-specific genes, *chitin synthase*, *phospholipid transporting ATPase (PPT-ATPase*, *vesicle-associated membrane protein 7B (VAMP)*, *plasma membrane-ATPase (PM-ATPase)* and *extended synaptotagmin-1 (ES-1)*, were highly expressed during the symbiotic association with *N*. *attenuata* (Fig 3B). We also checked the expression of five down-regulated Tags and their corresponding genes. For two of them (putative *ornithine decarboxylase* and *triacyl glycerol lipase)*, a weak down-regulation in response to infection could be confirmed, though levels were far less than expected based on the fold-change ratios obtained for the SuperSAGE libraries, and one gene (*cysteine synthase*) even showed the opposite effect while at the same time a putative *nuclear transport factor* and *myb-related protein 308* were unchanged (S4 Fig). Thus, it seems that the highly up-regulated genes are directly induced by AMF, and the down-regulated Tags are more dependent on other factors.

### Successful silencing of candidate genes by virus-induced gene silencing (VIGS) in *N*. *attenuata*

We selected two putative plant and fungal Tags that were highly expressed in response to infection to investigate their importance for the interaction. A putative *NaGH3*.*9* was selected because there is increasing evidence that auxins are important for successful fungal colonization [70], but details on the mechanisms are still scarce. *GH3*.*9* is an auxin-responsive gene functioning in auxin-based plant development [71]. The second plant gene selected encodes a putative germin-like protein (GLP). GLPs play a broad range of roles as enzymes, structural proteins, or receptors [72,73]. For *M*. *truncatula*, it was shown that *GLP* is expressed in arbuscule-containing cells [74], but it remains unknown whether *GLP* expression is essential for the interaction. Additionally, as a proof-of-principle, we constructed a VIGS vector for silencing *CCaMK*, because, in accordance with the results obtained for other species, stably transformed *N*. *attenuata* plants are not successfully colonized by AMF [51].

**Fig 4:**
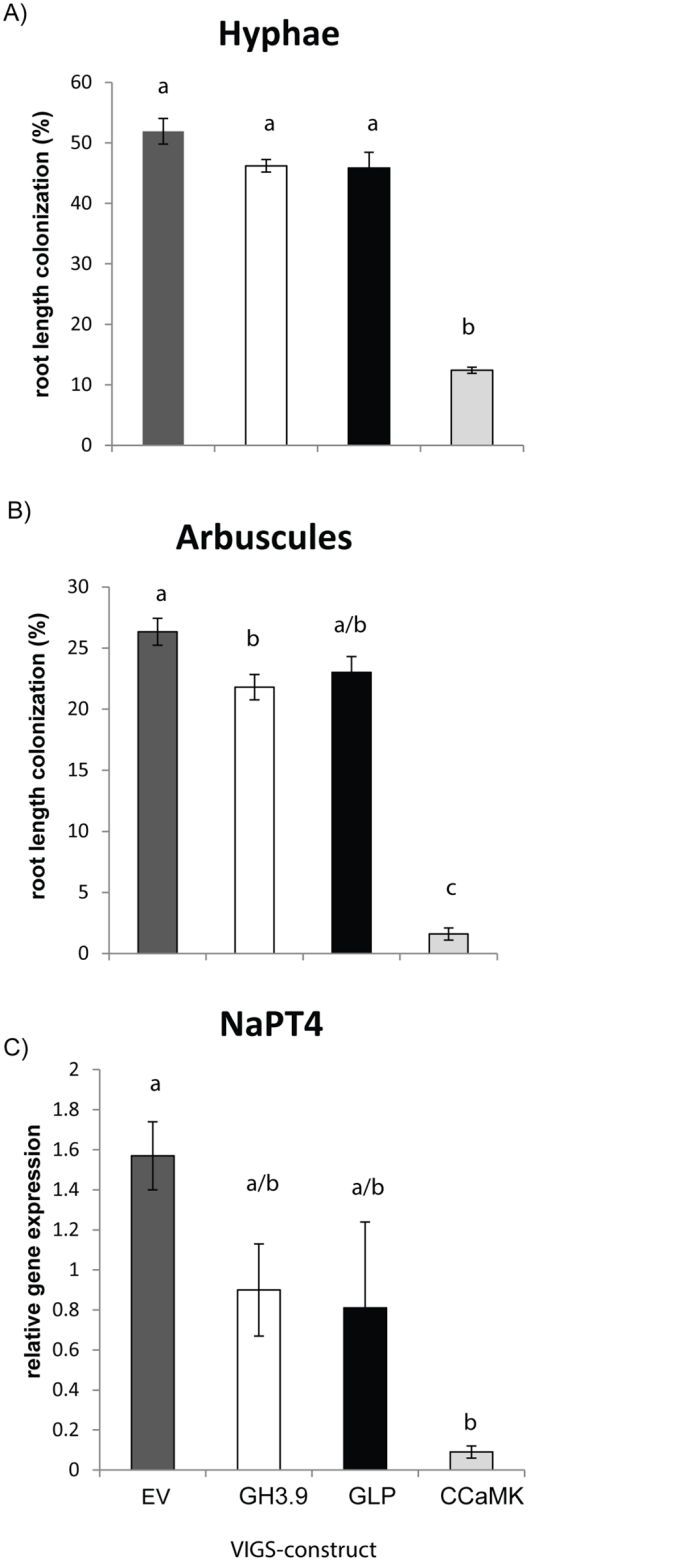
Root length colonization and expression of *NaPT4* of virus-infected control plants (EV) and plants silenced for *GH3*.*9*, *CCaMK* and *GLP* after inoculation with *R*. *irregularis*. *Nicotiana attenuata* plants inoculated with tobacco rattle virus vectors carrying a vector for silencing a *putative indole-3-acetic acid-amido synthetase* GH3.9 (GH3.9), a *germin-like protein* (GLP) and a *calcium and calmodulin dependent protein kinase* (CCaMK), and empty vector (EV) as control. Plants were harvested 28 days after inoculation with *R*. *irregularis*. A, B) Root length colonization by *R*. *irregulari*s was determined after Trypan blue staining with the gridline intersection method. Mean (± se, N = 5). C) *NaPT4* expression was determined by qPCR and normalized to *elongation factor alpha 1* (± se, N = 6). One-way ANOVA was followed by Tukey’s HSD, different letters indicate significant differences between the lines (*P* < 0.05). The VIGS constructs used for silencing are empty vector (EV) represented by dark gray bars, *putative indole-3-acetic acid-amido synthetase GH3*.*9 (GH3*.*9)*–white bars, *germin-like protein (GLP)*–black bars, *calcium and calmodulin-dependent protein kinase (CCaMK)*–light gray bars.

Virus-induced gene silencing of all three plant genes was successful, and plant growth was not affected (S5 Fig). The silencing efficiency was ~64% for *GH3*.*9* and *GLP* constructs and ~74% for CCaMK constructs (Table 2). Transient silencing of the *CCaMK* gene significantly reduced the number of hyphae and arbuscules compared to the number found in plants inoculated with the empty vector (EV) (Fig 4). qPCR analysis using *NaPT4*-specific primers supported the significantly lower number of arbuscules found for the microscopic analysis (Fig 4). Silencing *GLP* and *GH3*.*9* also reduced the infection parameters due to the variability among samples, but the effect was significant only for arbuscule formation in *GH3*.*9*-silenced plants (Fig 4B). Also auxin levels were not altered in *GH3*.*9*-silenced plants (S6 Fig). Interestingly, when a highly infective inoculum was used to colonize plants, 4 weeks after inoculation root length colonization and levels of *phosphate transporter 4* gene expression were not significantly different among empty vector controls and plants transiently silenced in *CCaMK* expression (S7 Fig).

**Table 2:** Selected genes for the functional characterization by virus-induced gene silencing (VIGS). Silencing efficiency is shown as the percentage of cDNA of the genes of interest in VIGS-silenced plants in comparison to empty vector roots measured by qPCR and normalized with *EF-1alpha*. *CCaMK—calcium and calmodulin-dependent protein kinase*, *GH3*.*9 –indole acetic acid-amido synthetase GH3*.*9*, *GLP—germin-like protein*,

Gene of interest	Vector	Silencing efficiency (%)*
*CCaMK*	pTVCCaMK	74%
*GH3*.*9*	pTVGH3.9	64%
*GLP*	pTVGLP	63%

*significant (p<0.05) difference compared to EV plants.

In addition to these plant genes, we also tried to silence genes of the fungal partner. Because previous attempts to silence genes of a heterotroph (insect larvae) using leaf material carrying gene sequences of the heterotroph by VIGS-treatment or using stably transformed plants were effective [45,75], we hoped for similar success. However, this approach was not successful for our plant-heterotroph (fungus) system, even when we used *MST2*, a fungal gene that has previously been successfully silenced using the *Agrobacterium rhizogenes* hairy root system in *Medicago truncatula* [46].

### *NaCCaMK* acts upstream of the putative *GH3*.*9* and *GLP*

**Fig 5:**
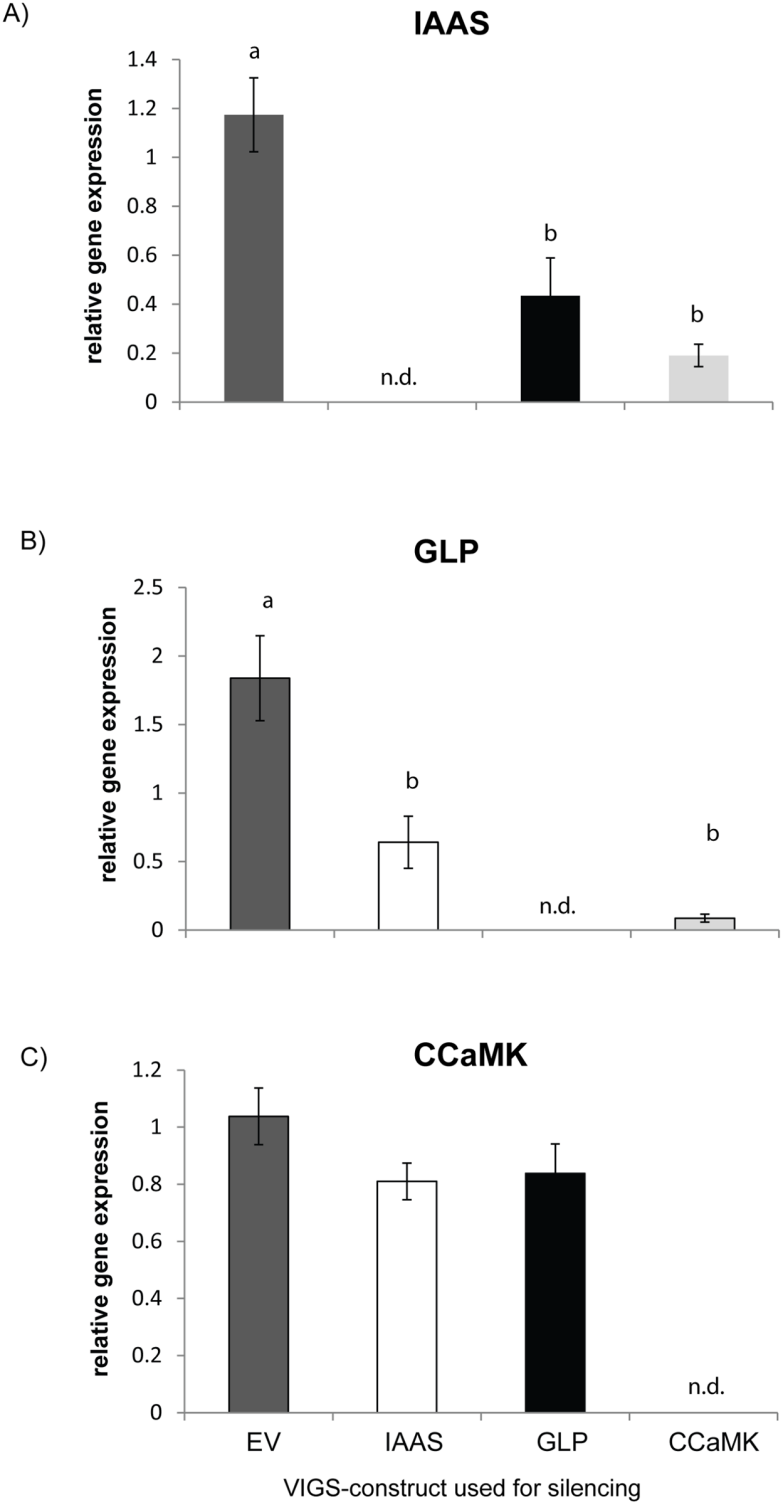
Transcript level of *GH3*.*9*, *GLP* and *CCaMK* analyzed by qPCR in virus-infected control plants (EV) and *GH3*.*9*, *CCaMK* and *GLP*-silenced plants. Plants were harvested 28 days after transfer to *Rhizophagus irregularis* inoculum, and relative transcript levels of A) *GH3*.*9* (*putative indole-3-acetic acid-amido synthetase GH 3*.*9*), B) *GLP* (*germin-like protein*) and C) *CCaMK* (*calcium and calmodulin dependent protein kinase*) in roots of the virus-inoculated plants carrying the different constructs were measured. *Elongation factor alpha 1* was used as a reference gene for normalization (± se, N = 6). One-way ANOVA followed by Tukey’s HSD, different letters indicate significant differences between the lines (P < 0.05). The VIGS constructs used for silencing are empty vector (EV) represented by dark gray bars, *putative indole acetic acid amido synthase GH3*.*9 (GH3*.*9)*–white bars, *germin-like protein (GLP)*–black bars, *calcium and calmodulin-dependent protein kinase (CCaMK)*–light gray bars.

In order to understand how the three plant genes are related with respect to each other after infection, we measured their expression in the different backgrounds. The transcript levels of *GH3*.*9* were strongly reduced in *NaGLP*- and *NaCCaMK*-silenced plants compared to EV plants (63 and 84%, one way ANOVA, *P* < 0.05 followed by Tukey’s HSD, Fig 5). Furthermore, transcript levels of *GLP* were found to be similarly reduced in *NaGH3*.*9* and *NaCCaMK* silenced plants in comparison to EV plants (65 and 95%, one-way ANOVA, *P* < 0.05 followed by Tukey’s HSD, Fig 5B), whereas the expression of *NaCCaMK* was similar in *NaGH3*.*9*- and *NaGLP*-silenced and EV-inoculated plants (one-way ANOVA, *P* = 0.204) (Fig 5C), indicating that *NaGLP* and *NaGH3*.*9* act downstream of *NaCCaMK*.

## Discussion

Here we used a SuperSAGE approach in combination with next-generation sequencing to characterize changes in gene expression after arbuscular mycorrhizal infection of *N*. *attenuata* roots. Unlike microarrays, this approach allowed us to explore genes that were not limited to those known from the transcriptome of *N*. *attenuata* and, additionally, to include fungal genes in the analysis. We used whole roots to obtain a global picture of the changes induced by AMF.

About 40% of the more than five million sequenced 26 bp Tags could be annotated with either *N*. *attenuata* or *R*. *irregularis* transcriptome; this number increased to 60% if only P-values of less than 0.05 were considered. The number is still lower than expected, but it has to be considered that the reference database is our in-house transcriptome database of *N*. *attenuata*. This database covers not the whole genome but, rather, only the genes expressed used for the generation of the library. Additionally, gene identification was based on 26 bp Tags; in these, sequencing errors more strongly influence the process of annotation than they do in longer reads, and next generation sequencing methods are known to be prone to sequencing errors that are substantially higher than the errors found in traditional Sanger sequencing [76,77]. Although the specificity of the 26 bp was high [38], it did not always match with a single gene; almost 20% of *N*. *attenuata* hits were multiple hits. Our study revealed changes not only in plant gene expression but also in fungal genes. About 10% of the annotated Tags—in total, 3698—matched with the *R*. *irregularis* transcriptome and 77% could be annotated with a putative gene function [34]. This number is higher than the number in the study [68], which found 762 fungal genes expressed during root colonization.

Many genes that were strongly induced in our study have also been shown in previous studies to be differentially regulated [29,68], indicating that a core set of genes is induced in plants in response to AMF infection independent of the plant species. This core group of genes cannot be related to a single defined function but, rather, relates to very different roles in a cell, such as transmembrane and vesicle transport, defense, signal transduction and catabolism (Fig 2A). These are the processes that mediate the reprogramming of cell metabolism in response to AMF-infection. We confirmed the up-regulation of *ABC-transporter A*, *GLP*, *cathepsin L*, *putative GH3*.*9* and *glutathione-S-transferase* after AMF infection in *N*. *attenuata* by qPCR; all of these are genes that have been shown earlier to be induced by AMF infection [23,28,30,68,78,79]. In contrast, the AMF-induced expression of an AB hydrolase has not been shown before, though other alpha / beta hydrolases, such as D14/DAD2, are known to be important for AMF infection [35,80]. Remarkably, among the phytohormone-related genes examined in the three studies compared here, auxin, ethylene and gibberellin-related genes are strongly induced during AMF infection in all three (S2 Table). *GA 20-oxidase* (GA20ox) genes belong to a gene family that converts C20-GA substrates through successive oxidative reactions to form C19-GA products [81], and if gibberellin signaling was impaired e.g. by a mutation in the DELLA protein expression, GA20ox was not induced by AMF and arbuscule formation was impaired [82]. This result suggests that the biosynthesis of active GA forms plays a role in arbuscule formation. *AP2-like ethylene-responsive transcription factors (AP2/ERF)*, whose expression was found to be enriched in the three independent studies compared here (S3), are defined in part by having domain-binding ethylene-responsive elements that are known to mediate nodulation factor signaling [83] and may also play a role in AMF signaling. For example, it was shown that the fungal effector protein SP7 interacts with ERF19 from *M*. *truncatula* [84]. The increased expression of genes involved in plant defense against pathogens and herbivores (Fig 2A), such as *PR-protein*, *S-norcoclaurine synthase*, *MLO-like protein*, *trypsin proteinase inhibitor* and *CCR4-associated factor 1* found after AMF infection of *N*. *attenuata*, *L*. *esculentum* and *Medicago truncatula* (S2 Table), may indicate the enhanced defense status of the infected plants; this enhanced status may lead to priming, which in turn may allow plants to react rapidly to attack [85,86].

Except for the *R*. *irregularis* sequencing project [34,55,87] and the 454-transcriptome sequencing of AMF-infected and non-infected tomato roots [68], few publications on fungal genes expressed during plant infection are available [27,46,88,89]. Based on node scores, genes involved in general metabolic processes strikingly dominated the annotated fungal Tags (Fig 2B). The observed pattern of fungal gene expression probably reflects the general metabolic activity for growth during the different developmental stages of the fungus within the root. Here, we confirmed the high expression of a *fungal plasma-membrane ATPase*, a *chitin synthase* and a *phospholipid transporting ATPase*, all of which have been shown earlier to be regulated during root colonization [27,29,55,89,90]. The two fungal genes *VAMP* and *extended synaptotagmin-1* have not been shown before to be highly expressed in mycorrhizal tissue; though the expression of *extended synaptotagmin-1* was induced after the treatment of *R*. *irregularis* hyphae with crude extracts of *M*. *truncatula* mycorrhizal roots [90]. For Arabidopsis, it was proposed that synaptotagmin-1 acts as a site for endoplasmic reticulum-plasma-membrane contact; this contact plays a role in the cellular adaptation of environmental stresses [91]. In *M*. *truncatula*, two highly homologous exocytotic VAMPs are required for formation of the symbiotic membrane interface in plant-AMF and plant-rhizobia interactions [92], and it can be assumed that the fungal VAMP plays a similar role in R. *irregularis* [93]. The expression of a putative fungal SEC13 protein was also up-regulated in infected roots. A *Lotus japonicus* homologue to the nucleoporin SEC13 protein plays a role when the rhizodermis is colonized by AMF, but not for the cortical endosymbiotic infection [94]. Although the function of the fungal gene is still unknown, it might be important for the early steps of root colonization. Further highly expressed putative fungal genes, such as *extended synaptotagmin-3*, *vesicle transport protein USE1*, *vesicle transport v-SNARE 12*, *protein transport proteins SEC1*, *SEC23*, *SEC61* and *yif1*, indicate the presence of strong vesicle trafficking, endoplasmic reticulum-plasma membrane interactions and high metabolic activity in the fungal cells, and suggest that Tags related to genes involved in cellular component biogenesis and development are enriched. We hypothesize that these genes are expressed and needed for the formation of hyphae and in particular for arbuscule formation.

In general, the fungal genes expressed during root colonization found in the present study overlap strongly with the genes expressed in the same species (*R*. *irregularis*) when it is colonizing tomato roots [68]. Based on the large number of significant changes in the transcriptomes found for *N*. *attenuata* and for *Medicago* and *S*. *lycopersicum* in resonse to AMF infection, an overlap of 18 genes with putative similar functions seems rather low, with more similar genes expressed in the two Solanaceous species [68] than in the legume [29,95] and in *N*. *attenuata*. In addition to the closer evolutionary relationship between tobacco and tomato compared to *Medicago*, the different experimental approaches (microarray vs. next-generation sequencing) may account for some of the differences. However, overall the data suggest that, in addition to a core set of gene functions expressed in the roots of all plant species in response to AMF infection (a response that is presumably of ancient origin), a large part of the gene expression pattern seems also to be species-specific and additionally influenced by environmental conditions and developmental stage. This hypothesis is supported by previous large-scale analyses that only showed little overlap or high variability even among different sampling years/seasons using the same species [28,96].

Gene-expression studies visualize only the pattern of genes expressed during the infection, whereas gene silencing enables a functional analysis of the differentially expressed genes to be carried out. Here, we used virus-induced gene silencing to further analyze the role of selected plant genes that were strongly induced during AMF infection. We successfully adapted the protocol established in our lab [43,66], and used *CCaMK*, a gene well-known to be important for AMF infection [15], for a proof-of-principle. We observed the expected strong reduction in arbuscule formation and *phosphate transporter 4* expression in response to gene silencing. As roots that developed before gene silencing were kept in a net and the remaining roots cut before transfer to inoculum, it was easy to harvest specific roots silenced for the gene of interest. The approach is similar to the approach used in the VIGS-silencing system established by [97], but allows for an increased root growth after gene silencing. Importantly, the strong reduction in root colonization and the reduced expression of *PT4* in *CCaMK*-silenced roots were observed only when the inoculum strength was moderate. When highly infected freshly cut leek roots were used—despite successful *CCaMK* silencing—root length colonization and the levels of *NaPT4* expression were similar in plants that had been inoculated with CCaMK and EV vectors, respectively. We infer that not only was the silencing efficiency (73%) insufficient to prevent colonization when roots were challenged with a highly active inoculum, but that also the roots of the cut leeks were functioning as hosts. The effect is probably similar to findings obtained with Arabidopsis, a plant species which usually cannot be infected by AMF. However, when Arabidopsis grows in close proximity to a highly AMF-infected *Medicago* host plant, *Arabidopsis* roots show typical AMF infection structures but no arbuscules [98]. Similarly, maize mutants impaired in the expression of an arbuscular mycorrhiza-specific phosphate transporter are not infected by arbuscular mycorrhiza when grown in single pots but are highly infected when grown together with infected chives and under field conditions (*trans*-complementation) [33].

Although the two *N*. *attenuata* genes, *GLP* and *GH3*.*9*, were successfully silenced, the effect on root colonization was not statistically significant. It remains unclear if silencing efficiency was not sufficiently high to observe an effect on AMF infection or if the genes do not play a crucial role for the infection process. However, the expression of the two genes was also significantly lower in *CCaMK*-silenced plants than in EV plants, indicating that they act downstream of *CCaMK*. This result is consistent with the finding that a *M*. *truncatula GLP1* showing 37% homology at the amino acid level with *NaGLP* was localized in the arbuscule containing cells [74]. The consistent up-regulation of an auxin-response factor in *N*. *attenuata*, *M*. *truncatula and S*. *lycopersicum* (S2 Table) and the high expression of a putative *GH 3*.*9* also in *Medicago* [95,99] and tomato [68,100] strongly suggest a role for auxin during AMF infection. This inference is supported by a previous study that showed an increase in conjugated auxins at late AMF developmental stages [101]. Furthermore, recent findings demonstrate that auxin production is needed for root colonization [102] and auxin perception for arbuscule formation [70], but how this phytohormone is involved in the interaction still needs further clarification.

Based on a recent study showing that tobacco rattle virus VIGS constructs expressing genes of heterotrophs in plants successfully silence those genes in heterotrophs when consumed [45], we extended our study to examine the plant-mediated RNAi potential for the AMF-plant interaction. However, this approach was not successful, and we infer that the periarbuscular membrane is a barrier that the viral RNA is unable to cross or that fungal RNAases are able to dismantle the silencing signal.

In conclusion, our work demonstrates that arbuscular mycorrhizal infection of plants induces a complex gene expression pattern with a highly conserved set of gene functions across different plant species. In addition to genes involved in phytohormone regulation, the increase in genes related to defense is striking and may prime plants to react more rapidly to future attack. However, a large portion of genes that respond to AMF infection do not do so consistently among different species and conditions, and context probably accounts for the variance seen in plant species, particular growth conditions and developmental stage. For an analysis of elicited gene function, we adapted a tobacco-rattle-virus-mediated gene-silencing protocol to silence genes involved in AMF interactions, allowing more in-depth studies of the roles of genes induced during AMF infection in roots.

## Supporting Information

S1 Fig Flow-chart showing the experimental approach used for the generation and analysis of the SuperSAGE libraries to find genes that are differentially regulated in the interaction between *Nicotiana attenuata* and *Rhizophagus irregularis*. Same-sized samples from infected and non-infected plants were harvested about 21 days after inoculation.
(TIF)

S2 Fig Fold-change distribution of significantly up- and down-regulated Tags (infected vs. non-infected, P<0.05). (TIF)

S3 Fig Kinetics of infection of *Nicotiana attenuata* roots by *Rhizophagus irregularis*. AMF colonization of roots harvested at different time-points after inoculation determined after Trypan blue staining according to [5959], and relative expression of *phosphate transporter 4* (*NaPT4*) determined by qPCR with gene-specific primers and normalized using actin primers as a reference. Each bar represents mean ± se (N = 5).
(TIF)

S4 Fig Validation of SuperSAGE Tags down-regulated in response to AMF infection by qPCR. (TIF)

S5 Fig Root and shoot biomass of plants inoculated with tobacco rattle virus carrying the following constructs: empty vector (EV), *probable indole-3-acetic acid-amido synthetase GH 9*.*3 (GH3*.*9)*, *germin-like protein (GLP)* and *calcium and calmodulin dependent protein kinase (CCaMK)*. N = 13±SE, one-way ANOVA followed by Tukey’s HSD, different letters indicate significant differences. If no letters are given, biomasses do not differ.
(TIF)

S6 Fig Auxin (IAA) levels are not altered in roots of virus control plants and indole-3-acetic acid synthase silenced plants. N = 6±SE.
(TIF)

S7 Fig A strong inoculum masks the effect of virus-induced gene silencing of *CCaMK* on infection by *R*. *irregularis*. Root length colonization by hyphae (A) and expression level of *phosphate transporter 4* (*NaPT4) (B)* in virus control plants (EV) and silenced plants (*CCaMK)* (73% silencing efficiency) were not significantly different from empty vector-treated plants (EV). Roots were harvested 35 days after transfer to fresh leek inoculum (infected roots and fungal spores on expanded clay particles). N = 6±SE for the microscopic analysis, n = 13±SE for gene expression analysis. *NaEF1alpha* was used as a reference gene for qPCR analysis. Different letters indicate significant differences. If no letters are given, differences were not significant.
(TIF)

S1 File Supporting Tables. **Table A**. List of primers used for the validation of SuperSAGE Tags related genes 35 days after inoculation with *R*. *irregularis*, **Table B**. List of primers used for the construction of VIGS vectors, **Table C**. Features of SuperSAGE libraries from *R*. *irregularis*-infected and non-infected *N*. *attenuata* root samples, **Table D**. Annotation of Tags with the in-house *N*. *attenuata* 454 transcriptome database [57], **Table E**. Fungal infection rates of roots used for A) SuperSAGE analysis and B) qPCR. Infection rates were determined after Trypan blue staining with the gridline intersection method according to [59]. For the SuperSAGE analysis, roots of 2 roots were pooled to one sample (N = 5); for the qPCR, 35-day-old samples grown on 10% inoculum were used (N = 4). **Table F**. Comparison of relative gene expression of selected genes for SuperSAGE and qPCR
(DOCX)

S1 Table Summarizing table characterizing the SuperSAGE Tags used in this study. (XLSX)

S2 Table Similar putative function of significantly up-regulated annotated plant genes overlapping in the present study, the study of Ruzicka et al.(2013) [63] with *S*. *lycopersicum* and the study of Hogekamp et al. (2011) [29] with *M*. *truncatula*. Putative functions found in all three studies (A), and in only two of the studies (B, C). The datasets contained the most highly up-regulated genes and their function for *N*. *attenuata* (log2 fold change >2, *P* < 0.05, 301 mapped and annotated Tags), AMF-infected tomato (*P* < 0.05, log2 fold change >2, 977 contigs, [68]), and *Medicago* roots log2 fold change >2, 394 gene IDs [29]).
(XLSX)

S3 Table Similar putative functions of annotated fungal tags and contigs significantly up-regulated after AMF infection found in the present study in *N*. *attenuata* and in tomato [68] (log2 fold change >2, p<0.05). (XLSX)
