# Supplementary figures and images for: Virus-Induced Gene Silencing Using Tobacco Rattle Virus as a Tool to Study the Interaction between Nicotiana attenuata and Rhizophagus irregularis
Source: PLoS One. 2015 Aug 20;10(8):e0136234. doi: 10.1371/journal.pone.0136234 (PMC4546398; doi:10.1371/journal.pone.0136234)

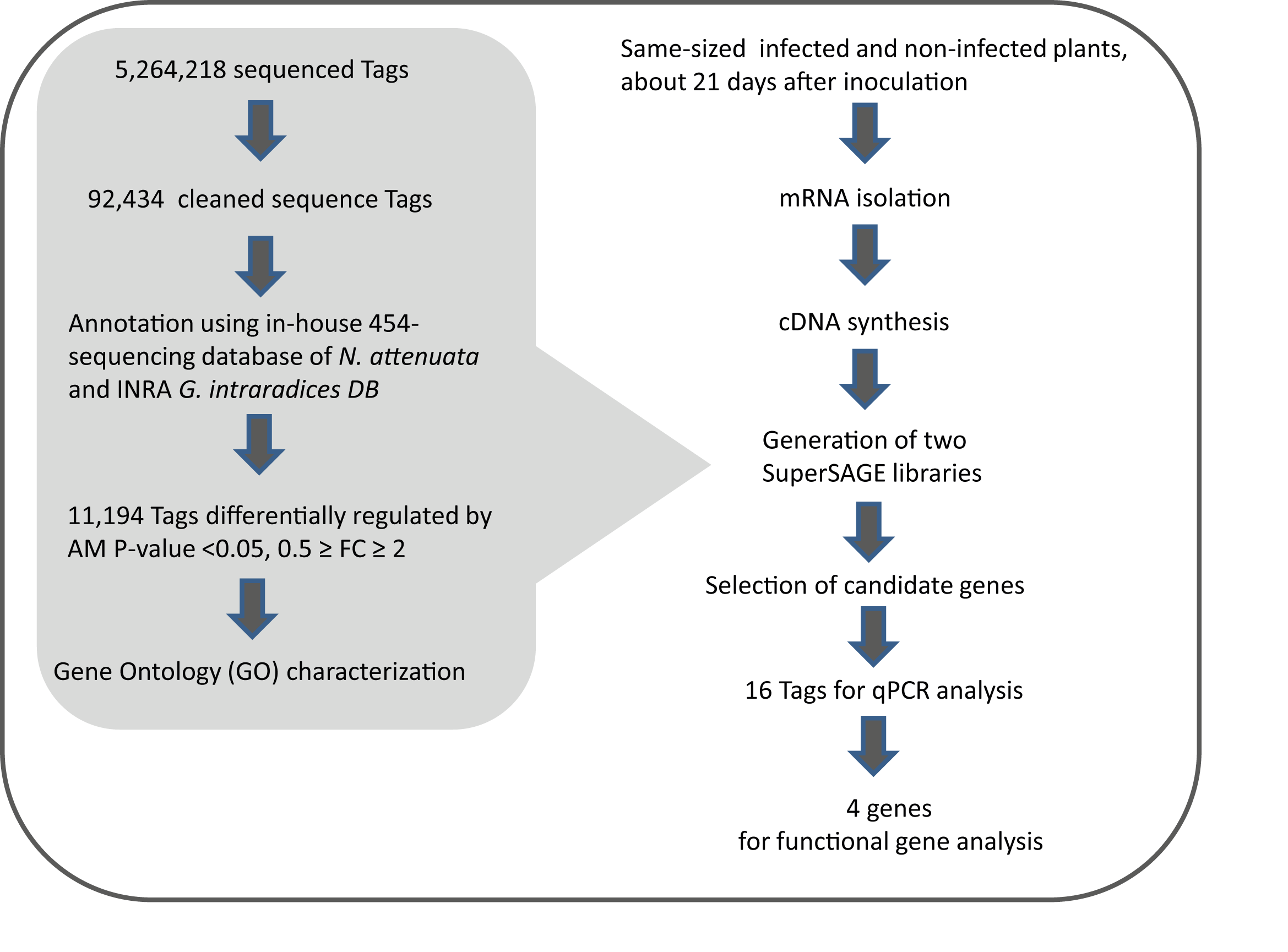

Supplement: S1 Fig — Same-sized samples from infected and non-infected plants were harvested about 21 days after inoculation. (TIF) [file pone.0136234.s001.tif]

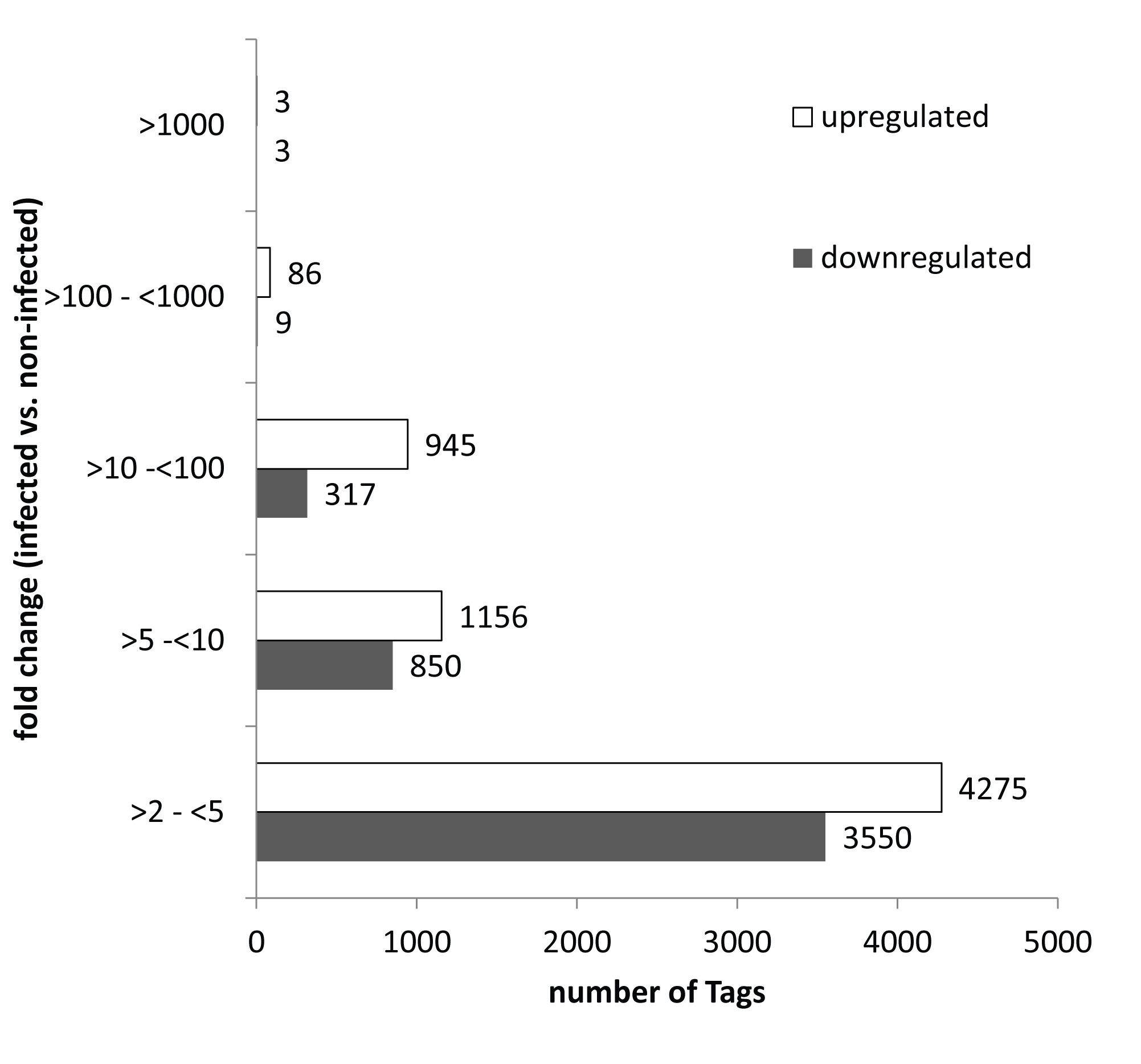

Supplement: S2 Fig — (TIF) [file pone.0136234.s002.tif]

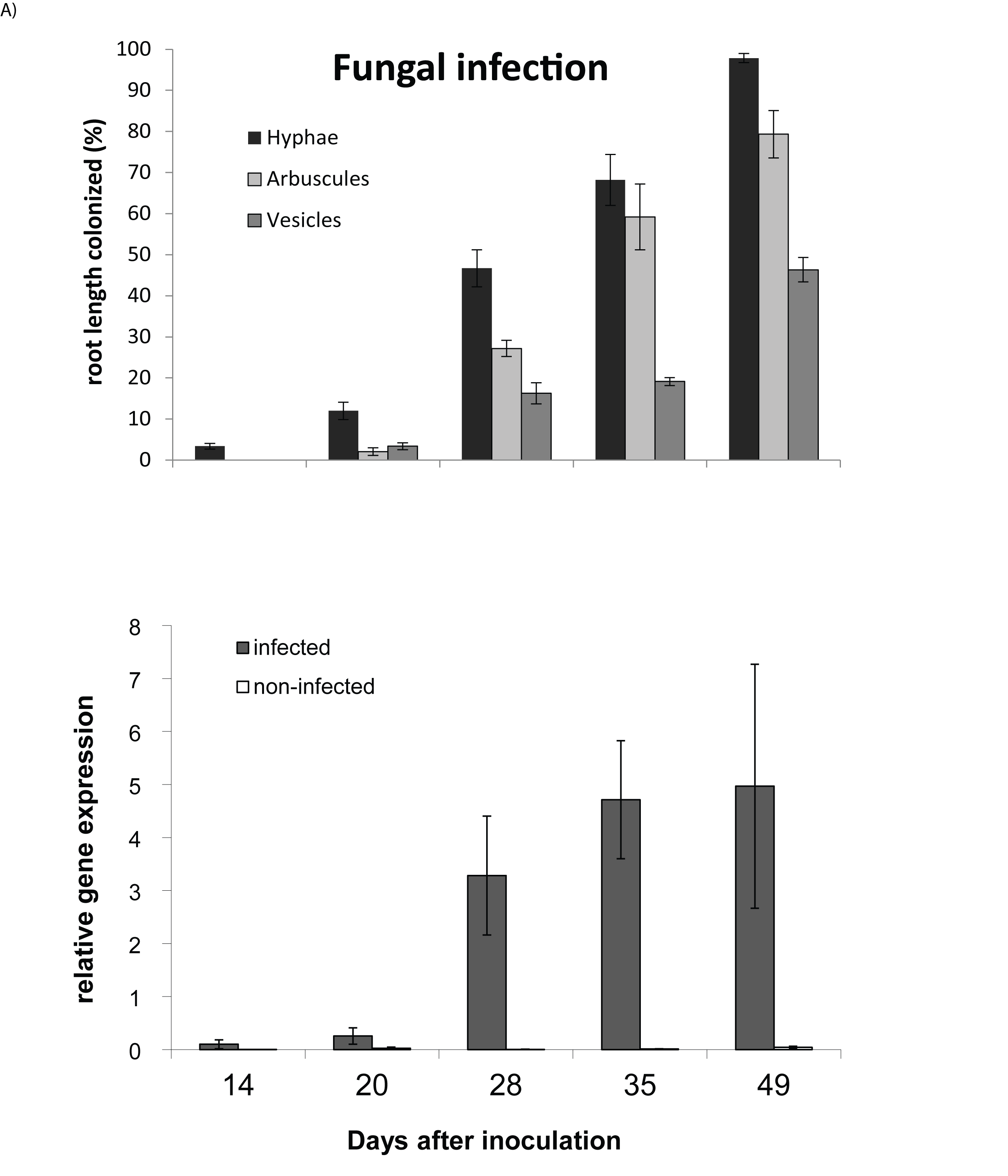

Supplement: S3 Fig — AMF colonization of roots harvested at different time-points after inoculation determined after Trypan blue staining according to [5959], and relative expression of phosphate transporter 4 (NaPT4) determined by qPCR with gene-specific primers and normalized using actin primers as a reference. Each bar represents mean ± se (N = 5). (TIF) [file pone.0136234.s003.tif]

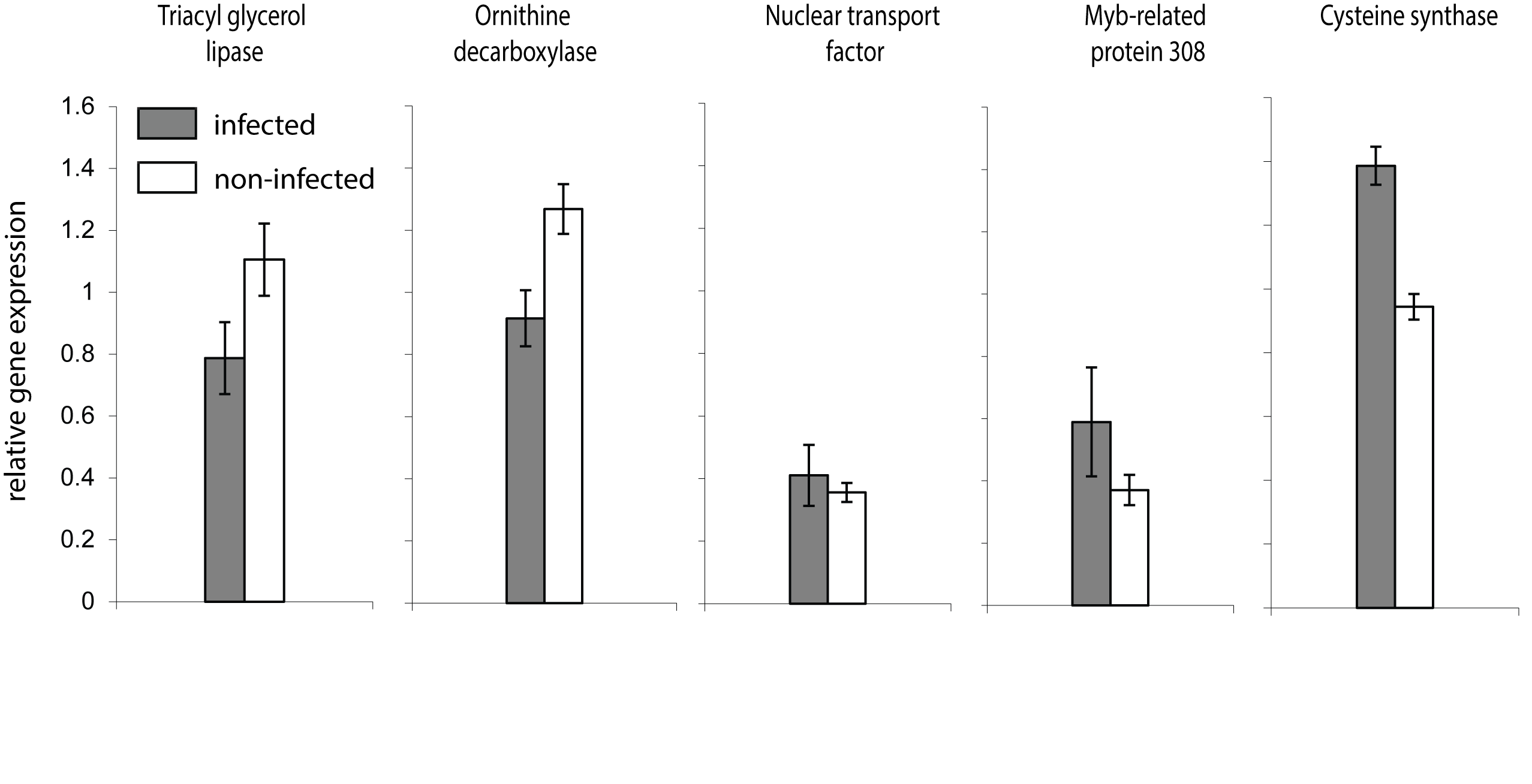

Supplement: S4 Fig — (TIF) [file pone.0136234.s004.tif]

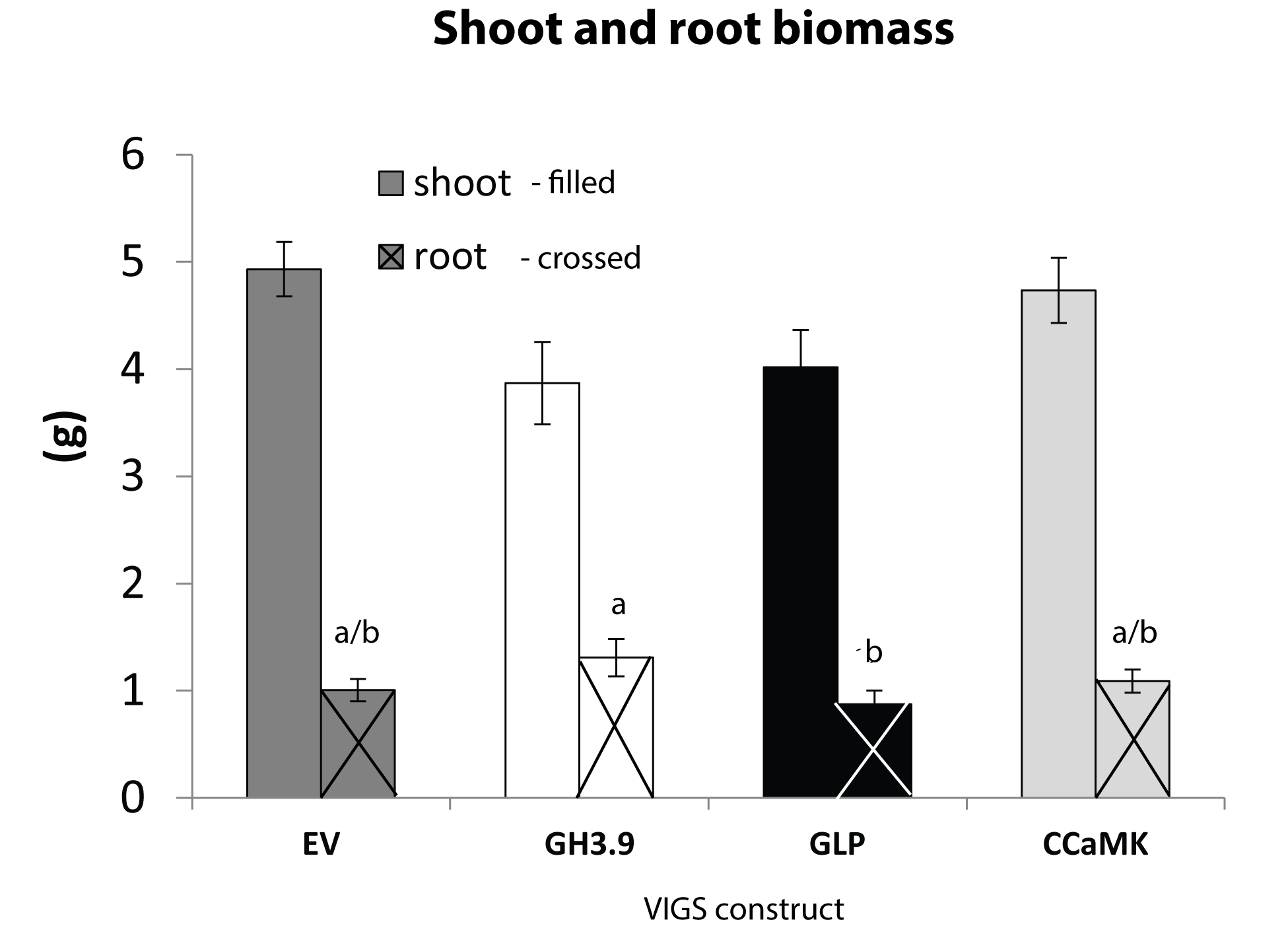

Supplement: S5 Fig — N = 13±SE, one-way ANOVA followed by Tukey’s HSD, different letters indicate significant differences. If no letters are given, biomasses do not differ. (TIF) [file pone.0136234.s005.tif]

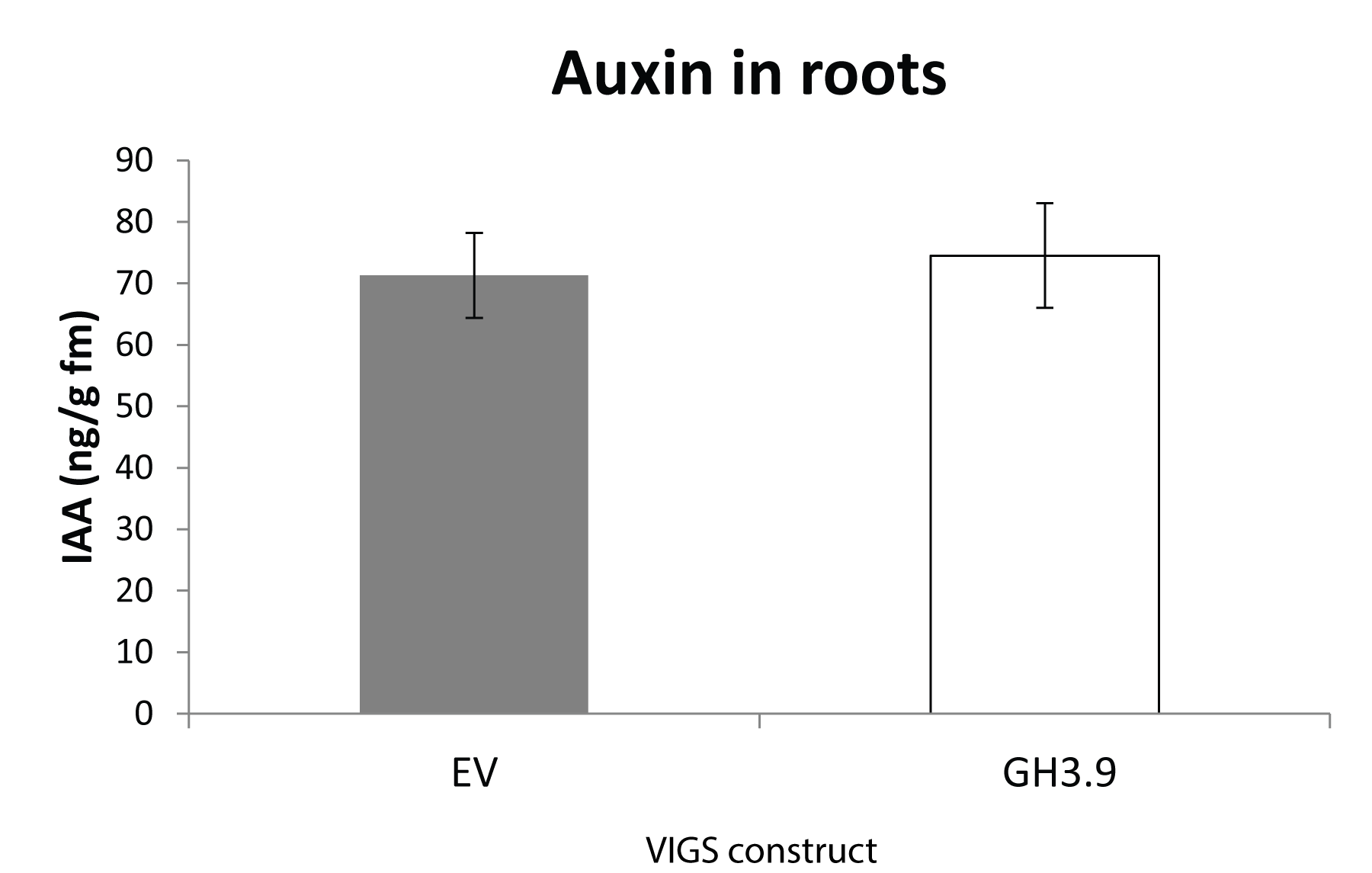

Supplement: S6 Fig — N = 6±SE. (TIF) [file pone.0136234.s006.tif]

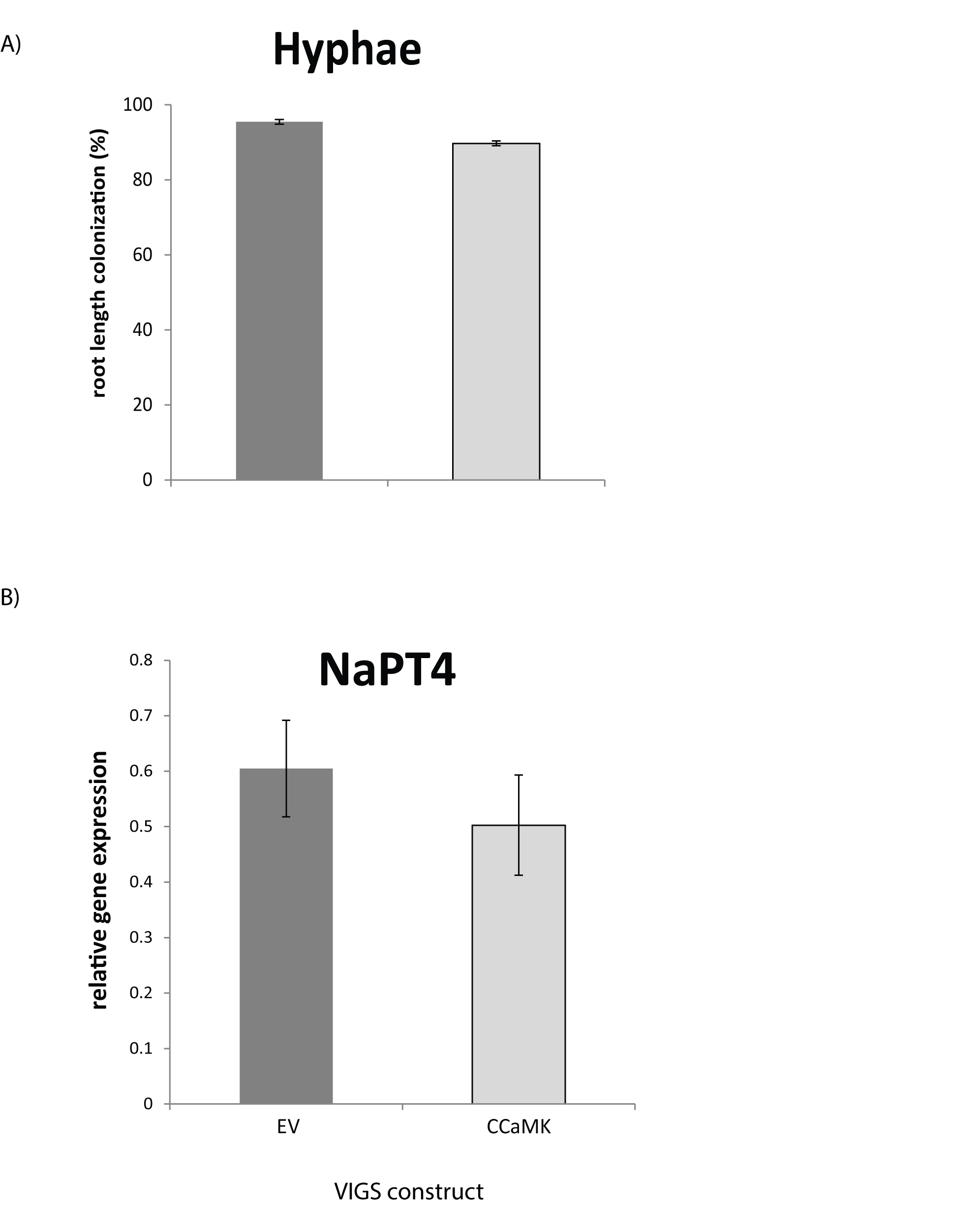

Supplement: S7 Fig — Root length colonization by hyphae (A) and expression level of phosphate transporter 4 (NaPT4) (B) in virus control plants (EV) and silenced plants (CCaMK) (73% silencing efficiency) were not significantly different from empty vector-treated plants (EV). Roots were harvested 35 days after transfer to fresh leek inoculum (infected roots and fungal spores on expanded clay particles). N = 6±SE for the microscopic analysis, n = 13±SE for gene expression analysis. NaEF1alpha was used as a reference gene for qPCR analysis. Different letters indicate significant differences. If no letters are given, differences were not significant. (TIF) [file pone.0136234.s007.tif]
