# Supplementary material for: Virus-Induced Gene Silencing Using Tobacco Rattle Virus as a Tool to Study the Interaction between Nicotiana attenuata and Rhizophagus irregularis
Source: PLoS One. 2015 Aug 20;10(8):e0136234. doi: 10.1371/journal.pone.0136234 (PMC4546398; doi:10.1371/journal.pone.0136234)
Supplement: S1 File — Table A. List of primers used for the validation of SuperSAGE Tags related genes 35 days after inoculation with R. irregularis, Table B. List of primers used for the construction of VIGS vectors, Table C. Features of SuperSAGE libraries from R. irregularis-infected and non-infected N. attenuata root samples, Table D. Annotation of Tags with the in-house N. attenuata 454 transcriptome database [57], Table E. Fungal infection rates of roots used for A) SuperSAGE analysis and B) qPCR. Infection rates were determined after Trypan blue staining with the gridline intersection method according to [59]. For the SuperSAGE analysis, roots of 2 roots were pooled to one sample (N = 5); for the qPCR, 35-day-old samples grown on 10% inoculum were used (N = 4). Table F. Comparison of relative gene expression of selected genes for SuperSAGE and qPCR (DOCX) [file pone.0136234.s008.docx]

S1 Table A: List of primers used for the validation of SuperSAGE Tags related genes 35 days after inoculation with *R. irregularis*

| **Tag-ID** | **GEO accession string** | **Putative *N. attenuata* genes** | **Primer F (5’-3’)** | **Primer R (3’-5’)** | | |
| --- | --- | --- | --- | --- | --- | --- |
| *SAGE_092030* | *Na_454_23440* | *ABC transporter A family member 3* | TGGATGAGCCCAGCACCGGA | TGCCTCATCCATCGAGTGTGTGGT | | |
| *SAGE_092078* | *Na_454_04923* | *AB hydrolase superfamily protein U17* | CCATGGCTTCCCTGAGCTTTGGT | GGTCAGGTGCCACAGCACGA | | |
| *SAGE_092353* | *Na_454_56637* | *Germin-like protein 9-3* | TGCGCGGGATCGTTCACAAAGT | GCGAGGGGTGTGTGTGAGGC | | |
| *SAGE_092101* | *Na_454_17784* | *Indole-3-acetic-acid amido -synthetase GH3.9* | CCACATGACCCAGCCAACCCG | GTCAGCCAGGTCCGACGGGA | | |
| *SAGE_092027* | *Na_454_93508* | *Glutathione-S-transferase* | TGCTTTGGGGTGCATTTTGGGT | GCCCATTTAACCAAGCCAGGGGT | | |
| *SAGE_092323* | *Na_454_53424* | *Cathepsin L1* | CCGAGTTAGATCATGGTGTCACGGC | CGCCCCAACTAGTTCCCCACG | | |
| SAGE_005566 | *Na_454_00105* | *Ornithine decarboxylase* | AAGTCGAACCGCTGCTCCGC | CGGCGTCTCCGCTTCCGATG | | |
| *SAGE_000204* | *Na_454_13199* | *Triacyl glycerol lipase* | GGCCAGCGCTAGAGACCAGG | GCACCTAGGCTGTGGCCAGT | | |
| *SAGE_000021* | *Na_454_08145* | *Myb-related protein 308* | TGGCTCAAAATGGGAAGGTCACCA | GGCCAGCAGCTTTAGGAAGAGACC | | |
| SAGE_004990 | *Na_454_01656* | *Cysteine synthase* | AGGAAGGCTTGCTTGTGGGCA | TCCCAGCATTTTCAGGGCGCTT | | |
| SAGE_000212 | *Na_454_05648* | *Nuclear transport factor 2* | AGCCATCGCCTACAGCGTGC | AGGTTAGCAAGCGCAGCCCG | | |
|  |  | *Phosphate transporter-4 [51]* | GGGGCTCGTTTCAATGATTA | AGCAGTGTAACGCCCTGTTT | | |
|  |  | *N.attenuata –elongation factor alpha-1 [*[*39*](#_ENREF_39)*]* | ACACTTCCCACATTGCTGTCA | AAACGACCCAATGGAGGGTAC | | |
|  |  | *N.attenuata CCaMK* | GCAAGAACAGATGGACG | TGCCCTGAACTTGCGCCG | | |
|  |  | *Nt-PT3* [[6](#_ENREF_67)9] | CACGACCTGAAGCTCAATTA | AGTCCATGACGACGAAGAAA | | |
|  |  | *Nt-PT5* [[6](#_ENREF_67)9] | TGGCGAATAGTATTGATGCT | ATGCTCTTGGCGTCACTAAC | | |
|  | ***Accession number*** | ***Putative R. irregularis genes*** |  |  | | |
| *SAGE_092293* | *Step3_c3080* | *Extended synaptotagmin-1* | CGATCAAGAAAGGCGAATTGAACCC | AGCTCCTTTTCTCCGTCAGTTACGA | | |
| *SAGE_091744* | *remain_c1562* | *Chitin synthase1* | TCGCGTCTGGTTGGCTCGTC | GTCGTGCTCACGTTCAGCCCA | | |
| *SAGE_090722* | *step3_c2140* | *Probable phospholipid-transporting ATPase* *C887.12* | CCCGATTACCGACCTCGCATGG | TTGCGCTTCTTGCCCAGATTCG | | |
| *SAGE_091840* | *step3_c583* | *Vesicle-associated membrane protein 7B* | TGTAGTCTTCGCATTCTTCGTCGGT | TGCGCAACAAAAGACCCCGAGT | | |
| SAGE_092044 | *step3_c2230* | *ATPase 3, plasma membrane-type protein* | TCCTGAAAGACGTGTTGAGAAA | ACCTGATAAATGGCGTCTTGGACAG | | |
|  |  | *R. irregularis -elongation factor 1 alpha [*[*46*](#_ENREF_46)*]* | ACGGACTTGATAACACCAACAGCGA | TCCAAACCGATGTGTGTTGAGGCT | | |
|  |  |  |  | |  |  |

S1 Table B: List of primers used for the construction of VIGS vectors

| Putative gene of interest | Forward primer  Sequence 5’ -> 3’ | Reverse primer  Sequence 5’ -> 3’ | Size of amplicon |
| --- | --- | --- | --- |
| *GLP* | GCGGCGGTCGACGCATCT GAACTCTTGTTCCTCG | GCGGCGGGATCCGCTTTAATCTTCTCAATGGTGCC | 354bp |
| *GH3.9* | GCGGCGGTCGACGGTTGAGTACACGAGTTTTGCTG | GCGGCGGGATCCACCCTGGAATCAAGAAGTTCCAC | 320bp |
| *CCaMK* | GCGGCGGTCGACGAAGAGGTCCTCAAAGCTATGG | GCGGCGGGATCCTTTACCATCACTATTTGCATCC | 348bp |
| *VAP* | GCGGCGGTCGACGGCTGATGATTCTTTTGGACGACG | GCGGCGGGATCCGGAAACCCACAAGCAGTACTGATG | 465bp |
| *PM-ATPase* | GCGGCGGTCGACGGTAATGATGATGCAGTTCATGCTG | GCGGCGGATCCGGAATAACTTGAGCGAACATCAG | 377bp |
| *MST2* | GCGGCGGTCGACGGAAGAGTTATTGCTGGCCTTGC | GCGGCGGGATCCTGCTTCTTCATCCCGATCATGATC | 299 bp |

S1 Table C: Features of SuperSAGE libraries from *R. irregularis-*infected and non-infected *N. attenuata* root samples

| Library | Infected | Non-infected | Total | (%) |
| --- | --- | --- | --- | --- |
| **Abundance classes of Tags*** | | | | |
| Very high-abundant: > 5,000 copies.million^-1^ | 7 | 4 | 11 | 0.01 |
| High-abundant: > 1,000 – 5,000 copies.million^-1^ | 70 | 103 | 173 | 0.19 |
| Mid- abundant: > 100 – 1,000 copies.million^-1^ | 1,364 | 1,480 | 2,844 | 3.14 |
| Low-abundant: < 100 copies.million^-1^ | 48,790 | 38,484 | 87,274 | 96.66 |
| Total | 50,231 | 40,071 |  | |
| **Copy number of Tags in abundance classes*** | | | | |
| Very high-abundant: > 5,000 copies.million^-1^ | 4.13× 10^4^ | 2.54× 10^4^ | 6.67× 10^4^ | 3.83 |
| High-abundant: >1,000 – 5,000  copies.million^-1^ | 1.21× 10^5^ | 1.83× 10^5^ | 3.04× 10^5^ | 17.45 |
| Mid- abundant: > 100 – 1,000 copies.million^-1^ | 3.30× 10^5^ | 3.81× 10^5^ | 7.11× 10^5^ | 40.81 |
| Low-abundant: < 100 copies.million^-1^ | 3.40× 10^5^ | 3.20× 10^5^ | 6.60× 10^5^ | 37.88 |
| Total | 8.32× 10^5^ | 9.1× 10^5^ |  | |

*values normalized to 1 million Tags

S1 Table D: Annotation of Tags with the in-house *N. attenuata* 454 transcriptome database [[56](#_ENREF_55)]

| No of matches | % | No. of Tags |
| --- | --- | --- |
| 26/26  24/26  20/26 | 35.5  44.5  51.3 | 32,808  41,172  47,416 |

S1 Table E: Fungal infection rates of roots used for A) SuperSAGE analysis and B) qPCR. Infection rates were determined after Trypan blue staining with the gridline intersection method according to [[59](#_ENREF_59)]. For the SuperSAGE analysis, roots of 2 roots were pooled to one sample (N=5); for the qPCR, 35-day-old samples grown on 10% inoculum were used (N=4).

**A**

| **Observation** | **average (%)** | **±SE** |
| --- | --- | --- |
| Hyphae | 74.7 | 0.89 |
| Arbuscules | 51.8 | 1.22 |
| Vesicles | 29.3 | 1.94 |

**B**

| **Observation** | **average (%)** | **±SE** |
| --- | --- | --- |
| Hyphae | 68.2 | 6.22 |
| Arbuscules | 59.2 | 8.00 |
| Vesicles | 19.1 | 0.96 |

S1 Table F: Comparison of relative gene expression of selected genes using SuperSAGE and qPCR

| Putative gene function | Log2 change  SuperSAGE | Log2 change  q-PCR |
| --- | --- | --- |
| ABC transporter A family member 3 | 4.5 | 2.0 |
| Germin-like protein (GLP) 9-3 | 6.7 | 7.8 |
| AB hydrolase superfamily protein U17 | 4.7 | 3.5 |
| Probable indole-3-acetic acid-amido synthetase GH3.9 | 4.8 | 7.8 |
| Glutathione S-transferase | 4.5 | 4.9 |
| Cathepsin L1 | 6.4 | 8.3 |
| Ornithine decarboxylase | -1.7 | -0.5 |
| Triacyl glycerol lipase | -3.6 | -0.5 |
| Myb-related protein 308 | -6.0 | 0.7 |
| Cysteine synthase | -1.7 | 0.6 |
| Nuclear transport factor 2 | -3.5 | 0.2 |
| Extended synaptotagmin -1* | 6.0 | 7.2 |
| Chitin synthase -1 | 3.8 | 7.7 |
| Probable phospholipid-transporting ATPase (PPT-ATPase)* | 2.7 | 8.4 |
| ATPase 3, plasma membrane-type (PM-ATPase)* | 4.6 | 3.8 |
| Vesicle-associated membrane protein (VAMP) 7B* | 4.0 | 6.4 |

*Genes related to *R.irregularis*
